# Truth-telling about suicide: Empowering Aboriginal and Torres Strait Islander people to engage with the media

**DOI:** 10.1177/00048674251328542

**Published:** 2025-03-27

**Authors:** Pat Dudgeon, Jemma Collova, Gerry Georgatos, Ee Pin Chang, Elizabeth Paton, Mel Benson, Megan Krakouer

**Affiliations:** 1 The Centre of Best Practice in Aboriginal and Torres Strait Islander Suicide Prevention, The University of Western Australia, Crawley, WA, Australia; 2 National Suicide Prevention and Trauma Recovery Project, Perth, WA, Australia; 3 Everymind, Newcastle, NSW, Australia

**Keywords:** Indigenous suicide prevention, media, Aboriginal Participatory Action Research, truth-telling

## Abstract

**Objective:**

Aboriginal and Torres Strait Islander voices are crucial when reporting on key issues facing Aboriginal and Torres Strait Islander peoples. To date, there has been very little representation of Aboriginal and Torres Strait Islander community members speaking to the media about suicide and mental health challenges, but this is changing. This paper outlines the process and outcomes of co-creating resources which aim to increase the confidence and capacity of Aboriginal and Torres Strait Islander peoples to talk to media about mental health challenges and/or suicide.

**Methods:**

Following an Aboriginal Participatory Action Research approach, this paper elevates the voices of 18 Aboriginal (Noongar) community members with lived experience of suicide and mental health challenges. This group took part in media training, led by a respected Aboriginal social justice advocate. Following the training, the group gifted stories about experiences engaging with the media, through research topic yarning.

**Results and Conclusions:**

The results from a thematic analysis identified a justified mistrust towards the media, a desire to speak up to the media, and the need for a range of resources to support truth-telling to the media. These results informed the co-creation of a guide to support truth-telling to the media, with the aim of building the capability of Aboriginal and Torres Strait Islander peoples to share their truth in a way which is safe for them.

Aboriginal and Torres Strait Islander voices are crucial to shifting the media’s deficit-focused narrative when reporting on key issues facing Aboriginal and Torres Strait Islander peoples, and to ensure truth-telling. The media can be a powerful tool for educating others about important issues, and can bring about meaningful change. However, despite being the focus of many media stories, Aboriginal and Torres Strait Islander voices are often not at the forefront of these stories.

The media has been responsible for perpetuating explicit and implicit racial biases against Aboriginal and Torres Strait Islander peoples, with negative impacts on health and wellbeing (Dudgeon et al., 2021; Fogarty et al., 2018; Larson et al., 2007). For example, the 1987 Royal Commission into Aboriginal Deaths in Custody found that *‘the racism, stereotyping of Indigenous peoples and inaccurate reporting of the media has a devastating impact on the lives of First Nations people and has caused distress and even suicide in some communities’*. Today, the mainstream media coverage of issues facing Aboriginal and Torres Strait Islander peoples can be skewed, downgrading, and ignorant (Thomas et al., 2019).

As the result of colonisation, one of the key issues facing Aboriginal and Torres Strait Islander peoples is high rates of suicide. Suicide rates for Aboriginal and Torres Strait Islander peoples are more than twice that of non-Indigenous peoples (Kreisfeld and Harrison, 2016), with evidence that this rate is increasing (AIHW, 2023). Aboriginal and Torres Strait Islander peoples and communities have the solutions needed for effective suicide prevention (Dudgeon et al., 2016; Sjoblom et al., 2022). For example, Aboriginal and Torres Strait Islander peoples have long advocated for community-led, place-based strategies, which focus on strengthening social and emotional wellbeing (Human Rights and Equal Opportunity Commission, 1997; Dudgeon et al., 2016). These knowledges are starting to be incorporated into national health policy (Commonwealth of Australia, 2017, 2021). However, Aboriginal and Torres Strait Islander peoples and communities often receive little space in mainstream media to share lived experiences of suicide and mental health challenges, as part of truth-telling. When issues impacting Aboriginal and Torres Strait Islander peoples are given space in mainstream media, they are usually covered negatively (Stoneham et al., 2014) or in ways that delegitimise Aboriginal and Torres Strait Islander perspectives (Thomas et al., 2019). Further, some Aboriginal and Torres Strait Islander peoples report a lack of confidence and skills to have these conversations (Heard et al., 2022).

It is important that Aboriginal and Torres Strait Islander peoples are empowered to confidently talk to the media about mental health challenges and suicide, as part of truth-telling. Truth-telling can include sharing lived and living experiences of suicide, advocating for better support, sharing unmet community needs, or sharing what is (and is not) working in a community. Truth-telling is important as it can help to change the media’s deficit-focused narrative when reporting on these issues, and to ensure that lived-experience voices are heard. Sharing one’s truth is also particularly important from a suicide prevention perspective. Truth-telling plays an important role in healing from the impacts of intergenerational trauma (Dudgeon et al., 2024b; Wilmott et al., 2024) and can reassert self-determination (Barolsky and Berger, 2023). Sharing experiences of overcoming a crisis can also reduce suicide risk, and elevating the voices of lived experience can empower others to overcome difficulties (Niederkrotenthaler et al., 2010, 2022), as seen in non-Indigenous contexts.

This paper outlines the process and outcomes of co-creating resources which aim to increase the confidence and capacity of Aboriginal and Torres Strait Islander peoples to talk to the media about mental health challenges and/or suicide. In particular, this paper elevates the voices of Aboriginal (Noongar) community members with lived and living experiences of suicide and mental health challenges. The paper outlines community member’s (1) perceptions of mainstream media, (2) resource needs and (3) recommendations for the co-creation of such resources. The results from this paper have informed the co-creation of a Fact Sheet,^1^ focused on truth-telling to the media. We use the term ‘co-create’ to emphasise the high level of involvement, continuous and active input, and position as value creators, of the Aboriginal community members (Vargas et al., 2022). This involvement occurred from project conceptualisation to resource development.

## Method

### Research approach

This research was guided by principles of Indigenous standpoint theory (Nakata, 2007). Indigenous standpoint theory involves enquiry which recognises Aboriginal peoples as the experts of their own lived experiences, and which privileges these worldviews throughout the research process (Foley, 2006). This approach challenges the notion of the researcher as the ‘knower’, and the participant as a subject of investigation, and aligns with decolonial practices used here (Dudgeon et al., 2024a; Dudgeon and Walker, 2015; Fogarty et al., 2018; Smith, 2021 [1999]).

The project adopted an Aboriginal Participatory Action Research (APAR: Dudgeon et al., 2020) approach. APAR empowers Aboriginal peoples to describe their own reality and promotes community ownership over research processes, ensuring that the research outcomes are relevant to Aboriginal peoples and communities. In line with this approach, the resources from this project were co-created with Aboriginal community members. The resources aim to empower Aboriginal peoples share their truth to the media, in a way which is safe for them. This focus was identified as a priority by the Aboriginal peoples involved in the project, and validated by the community members. Phrases such as *‘truth-telling to media’* and *‘sharing one’s truth’* are used in this paper to reflect the language used by the Aboriginal community members.

The Centre of Best Practice in Aboriginal and Torres Strait Islander Suicide Prevention (CBPATSISP) led this project, and had oversight and control over all significant project decisions (governed by P.D.), ensuring Indigenous governance. The project occurred in collaboration with Everymind, who provided financial and project support. The CBPATSISP selected a highly experienced Aboriginal consultant with extensive experience engaging with the media about social justice issues (M.K.). The project was supported by two non-Indigenous CBPATSISP research staff with experience working in culturally safe ways (J.C. and E.C.), and a non-Indigenous person who was invited to contribute by the Aboriginal governance team based on their media expertise and community reputation (G.G.). Non-Indigenous staff from Everymind (M.B., E.P.) also supported the project.

The project adhered to NHMRC principles for ethical research with Aboriginal and Torres Strait Islander peoples (National Health and Medical Research Council, 2018), and received ethics approval from an Aboriginal health ethics committee (WAAHEC HREC1144). The research process and APAR methodology aimed to support culturally safe and empowering experiences (Milroy et al., 2022).

### Participants

Eighteen Noongar community members (19–65 years) attended the media training, invited by the Aboriginal media expert. This was a very unique group, with deep knowledges gained through lived and living experiences of mental health challenges and/or suicide. Of those who attended, 13 community members had never spoken to the media (nor been approached), 1 had been approached by the media but decided not to speak, and 4 had spoken to media at least once about mental health challenges and/or suicide.

The Aboriginal media expert was highly respected and trusted by the community, with extensive experience supporting Aboriginal and Torres Strait Islander peoples in a trauma informed way. They had personal connections and relationships with the community members, with trust built over several years. These characteristics were essential to convening the group, ensuring the cultural safety of the attendees during the training, and informing the development of resources.

### Measures

#### Quantitative and qualitative survey

A survey was co-designed between the Aboriginal media expert and CBPATSISP/Everymind staff. The aim of the survey was to understand the extent of experience the Aboriginal community members had talking to media, and to evaluate the impact of media training. Participants responded to three questions: (1) Do you feel you know how to engage with media to talk about mental health or suicide, (2) How likely would you be to engage with media to talk about mental health or suicide, and (3) How confident do you/would you feel talking to media about mental health or suicide? Participants responded to these questions before the media training, and immediately after the training, on a 7-point Likert-type scale ranging from 1 (not at all) to 7 (very). Participants also answered a question which asked about their biggest worry when talking to the media (see Supplemental Appendix 1 for full survey).

#### Focus group yarns

The focus group yarning schedule was co-designed between the Aboriginal media expert and CBPATSISP/Everymind staff. There were six key questions to guide the yarn, and three additional questions if time permitted (Supplemental Appendix 2). The aim of the focus group was to understand how attendees benefitted from the media training, and to get a more nuanced understanding of resource and support needs.

### Procedure

**Figure 1.**
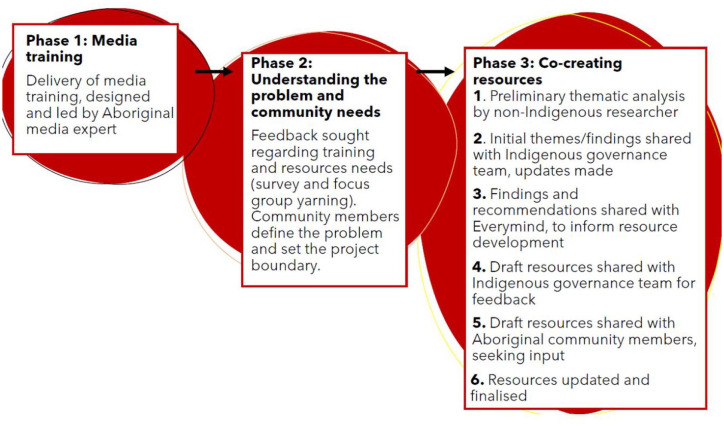
The three-phased approach to the project.

This project occurred in a three-phased approach (see Figure 1). Phase 1 involved the delivery of media training, in order to clarify resource/support needs. Phase 2 aimed to get a deeper understanding of the problem and resource needs. Feedback revealed that additional resources (beyond the training) were also necessary. Phase 3 involved cultural validation and a final feedback loop for the finalisation of the co-created resources/recommendations. Quantitative and qualitative data were gathered in Phase 2, and validated through the feedback loop in Phase 3.

#### Phase 1. Media training programme

As a first step, team members from the CBPATSISP and Everymind met with the Aboriginal media expert to discuss the project. The Aboriginal media expert then developed the media training, which aimed to build confidence in Aboriginal peoples to share their truth about mental health challenges and suicide with the media. The training was delivered on Whadjuk Noongar Country (Perth, Western Australia), as a full-day event. The training focused on topics including Understanding the mechanics of media; Ensuring the best possible advocacy on suicide awareness and prevention; Tips and tools for engagement; and Having confidence to build media relationships.

The session was run at a venue selected by the Indigenous governance team, due to its inviting space that looked out to nature. The session started with an acknowledgement of Country, and introductions by each person. The Aboriginal media expert led the session, with support from the non-Indigenous researchers and Everymind staff. Morning tea and lunch were provided. Attendees provided informed written consent prior to participating.

#### Phase 2: understanding the problem and resource needs (data collection)

Participants individually completed a short survey (pre- and post-training) and collectively participated in a focus group yarn after the training (see Materials and Supplemental Appendix 1 and 2). Two focus groups were run (about nine people in each group) to allow for meaningful discussions. The initial plan was to have one focus group facilitated by the Aboriginal media expert; however, more people attended the training than expected. Therefore, the Aboriginal media expert facilitated one focus group, and a non-Indigenous researcher facilitated the other focus group with support from the Everymind staff. As part of the focus group, the community members shared their knowledges through yarning. Yarning is a way that Aboriginal peoples refer to sharing knowledge, that draws upon cultural protocols and ways of being. Yarning is also a powerful methodology used in research with Aboriginal peoples (Bessarab and Ng’Andu, 2010; Kennedy et al., 2022). A mix of social yarning and research topic yarning were used to address the research questions (Kennedy et al., 2022). Each focus group collaboratively discussed the questions, with conversations flowing naturally. The yarning was recorded for note checking, and summary notes were taken by the research staff. Each person was provided a gift-voucher after completing the training and another after participating in the yarn.

#### Phase 3: co-creating the resources (data analysis and validation)

Phase 3 involved the collaborative analysis of feedback from Phase 2, to inform the co-creation of the resources. The analytical approach combined APAR (Dudgeon et al., 2020) with reflexive thematic analysis (Braun and Clarke, 2021), Figure 1. A non-Indigenous researcher (J.C.) qualitatively analysed data from Phase 2, with input from the Indigenous governance team. The qualitative survey data and focus group data were analysed together, using thematic analysis (Braun and Clarke, 2006). The researcher first immersed themselves in the data, coding the qualitative data through an inductive approach. When coding, the researcher focused on understanding the (1) resource needs and (2) attitudes towards the media. These results were summarised into a report, with draft recommendations.

Following an APAR approach, preliminary results were first shared with the Aboriginal media expert for early input into themes. The results were then shared with the senior Aboriginal researcher, for further validation. The updated results/report was then shared with the Everymind team. Based on these resources, Everymind drafted the Fact Sheet. Members of the CBPATSISP team (P.D., J.C.) then met with the Aboriginal media expert (M.K), and non-Indigenous media expert (G.G.), to review and further refine the draft resources.

A smaller group (*N* = 5) of community members who attended the training were invited to take part in a feedback session, selected based on their unique lived experiences, and considering gender/age. During this process, the community members yarned about the resources, and went through the results and recommendations through a relational process led by the Aboriginal media expert. The results and recommendations were summarised in print format (with engaging visuals and colours). Each person was provided a printed copy, which they could refer to during the yarn, and keep afterwards. Each person was reimbursed with a gift voucher, and shared a catered lunch together.

The results, draft report, and resources were updated based on the feedback from the above session. For example, there were changes to wording to align with how the community members described their own lived experiences, and additions to ensure culturally sensitive language. Some people also gave permission for us to share stories of their experiences with media.

## Results

### Survey results (quantitative)

As a first step, we analysed the quantitative results from the survey to understand the impact of the media training. After completing the training, the Aboriginal community members reported knowing more about how to engage with the media (*M* = 4.1) as compared to before the training (*M* = 2.9), *t* = 4.1, *p* < .001. Community members were also more likely to engage with media after the training (*M* = 4.1) as compared to before the training (*M* = 3.1), *t* = 2.9, *p* = .006, and felt more confident to talk to media after the training (*M* = 4.0) as compared to before (*M* = 3.1), *t* = 2.8, *p* = .005.

### Thematic analysis (qualitative)

As mentioned above, the qualitative survey data and focus group data were analysed together, using thematic analysis (during Phase 3). The final results are outlined below, and were used to inform recommendations (Table 1) for the co-creation of resources.

**Table 1.** Recommendations following the media training.

#	Recommendation
1.	The development of resources which empower Aboriginal and Torres Strait Islander peoples to speak up to media about mental health challenges and suicide, and engage in truth-telling to educate the nation. These resources should balance the need for truth-telling as part of healing, with the need for safe reporting of suicide. These resources could include: 1. A Fact Sheet / pamphlet, co-created with Aboriginal and Torres Strait Islander peoples, and developed under Indigenous governance. This resource should include tips on understanding the media, and how to confidently engage with media to share lived experiences and/or for advocacy purposes. (a) This resource could include current statistics/facts about Aboriginal and Torres Strait Islander mental health challenges and suicide, which Aboriginal and Torres Strait Islander peoples can draw upon when talking to media. (b) This resource should be easy to understand, visually engaging, and strengths-based. (c) This resource should consider the cultural and linguistic diversity of Aboriginal and Torres Strait Islander communities. 2. Videos co-created with Aboriginal and Torres Strait Islander peoples, and developed under Indigenous governance. (a) Videos should feature Aboriginal and/or Torres Strait Islander peoples (of varied ages), sharing their stories about how they spoke to media about mental health challenges and suicide, and providing tips on effective engagement and truth-telling (b) This resource should be easy to understand, visually engaging, and strengths-based (c) This resource should consider the cultural and linguistic diversity of Aboriginal and Torres Strait Islander communities.
2.	The development and/or delivery of any future Indigenous media training should: (a) Empower Aboriginal and Torres Strait Islander peoples and communities, especially young people, to speak up to media when talking about mental health challenges and suicide (b) Be developed under Indigenous governance, (c) Be designed by Aboriginal and Torres Strait Islander peoples, (d) Be delivered by a trusted and respected Aboriginal and/or Torres Strait Islander person who can work in a culturally safe way with communities.
3.	Non-Indigenous media workers need to be culturally responsive and receive training on how to work with Aboriginal and Torres Strait Islander people and communities, when reporting on Aboriginal and Torres Strait Islander mental health challenges and suicide.

#### A distrust towards the media

There was a justified distrust towards the media. The Aboriginal community members shared stories of the media twisting the truth, being deficit-focused, perpetuating negative stereotypes, not listening to community/families, and blowing stories out of context. The group discussed how the media can be useful to help share a story and bring about change, however, sometimes the media can stretch the truth to benefit themselves. The group agreed that the media did not always tell the whole story. For example, the media might focus on a single part of a story, without talking about the true challenges experienced by the community (e.g. social determinants of health). When asked, *‘what do you want to see in media reporting?’*, one member responded that the media *‘need to tell the truth for a start’*, and *‘the sensitivity is no good . . . They’ve got no respect . . .’*.

The Aboriginal community members expressed that the media did not understand cultural protocols, and did not always work in culturally safe ways. For example, an Aboriginal community member shared a story of the media posting photographs of a family member who had taken their life, without permission on the news. There are also cultural protocols regarding the use of names for those recently deceased, and permission should be sought from families to use names. The media was also seen to infringe on basic Human Rights, seeming to ignore the tenet to ‘do no harm’. Taking already traumatic events out of context and portraying these in sensationalising ways contributed to re-traumatisation. For example, a group member shared a story where they received over 600 phone calls from the media immediately following a family suicide, without any consideration of this impact on their grief and wellbeing.

The group discussed the need for a larger Indigenous workforce in the media, and more experienced non-Indigenous peoples who worked in culturally safe ways and who took time to understand the context of the stories. The media needs to be trained on how to work with Aboriginal peoples and get the story right.

#### A desire to speak up and speak out to the media

The group expressed a desire to build confidence to talk to the media, such as through the training they attended, and expressed that this training should be available for the wider community. Communities should be empowered to speak out and speak their truth. The group viewed it as important to have a wide range of Aboriginal voices, and saw the voices of many as powerful:

*A lot of people want to speak out, and need to speak out, but they don’t have avenues and that . . . Everyone has stories that they need to tell.*

*It’s good for our community because not all our mob are able, capable . . . shy and shame, and all that stuff, to have people speak . . . We need a wide range of voices, because the voices of many is powerful. But our whole community needs people talking*.

There was also a particular focus on the need to empower young people to speak to the media. Young people should learn how to share their lived experiences, to build their confidence for the future.

*They’ll need to speak up for themselves one day.*

*When we are talking about issues that relate to young ones . . . why are we talking about them all the time? We should elevate them to speak . . .*

Not speaking out was identified as detrimental to mental health. In contrast, the Aboriginal community members identified the process of talking about mental health challenges and suicide, and sharing their lived experiences in a culturally safe space, as a healing experience. It was seen as empowering to share one’s story in the way that one wants to tell it:

*A lot of our mob keep a lot of stuff bottled up, especially our young ones. And that’s where we see suicide, suffering in silence.*

*I find, myself, talking about it, speaking about it as healing.*

*Needs to be talked about more.*

This training empowered the community members to speak to the media. Some people felt that during emotional events (e.g. during bereavement), the media should speak to an Aboriginal community spokesperson, rather than directly approaching family. The media should approach a person who the family has specifically nominated.

#### A range of resources are needed

When asked about resources which would be useful, attendees suggested a booklet/pamphlet that they could take with them and share with communities. This resource should be eye-catching, colourful, and with artwork developed by community artists. This resource could contain tips for how to engage with media when truth-telling and sharing lived experiences, and include facts about Aboriginal and Torres Strait Islander mental health challenges and suicide.

The group saw value in hearing stories, and learning from a range of Aboriginal people who have experience talking to the media. Some group members also spoke about the value of video resources, which would be particularly valuable for people for whom English is a second language. These videos should feature relatable people (of varied ages), speaking lay language, and sharing their stories about their engagement with the media. The group also spoke about the value of yarning circles, coming together, and sharing stories to facilitate learning and healing.

In the context of any future media training, the group discussed the importance of the facilitator in creating a safe space. It would be essential to have a facilitator who is understanding and supportive, and who can break *‘language down black-fulla way and white-fulla [way]’*.

### Recommendations

Based on the above findings, the following recommendations were co-developed (Table 1).

## Discussion

The media has the power to educate people about important social issues, and influence change. However, not all voices are given equal opportunity to share their truth. In this paper, we outline the process and outcomes of co-creating resources with Aboriginal community members, which focus on facilitating truth-telling to the media in a safe and supported way. These resources were co-created with Aboriginal community members, with the aim of building the capacity of Aboriginal peoples to share their lived experiences and truth about mental health challenges and suicide. Ultimately, these resources will help to ensure that Aboriginal and Torres Strait Islander voices are at the forefront of stories about Aboriginal and Torres Strait Islander peoples.

The stories gifted by the group highlighted a justified mistrust towards the media, and a desire for truth-telling about mental health challenges and suicide to the media. From these stories, it was clear that the media did not always work in culturally safe ways. Nevertheless, the group acknowledged the importance of speaking to the media, and in particular, the need for young people to do so. Engaging in truth-telling (Wilmott et al., 2024) and sharing lived experiences of overcoming a crisis (Niederkrotenthaler et al., 2010, 2022) can reduce suicide risk and promote healing (Dudgeon et al., 2024b). In this context, truth-telling to the media can also be considered suicide prevention (Dudgeon et al., 2016), as long as these stories are received and shared in culturally safe ways.

This paper outlines recommendations intended to guide the development of future resources to facilitate Aboriginal people to engage with the media. Adopting these recommendations, Everymind and the CBPATSISP co-created a Fact Sheet in order to support truth-telling about mental health challenges and suicide to the media. Importantly, in line with APAR (Dudgeon et al., 2020), the Aboriginal community members were involved from project/resource conceptualisation through to resource development. This process allowed for the co-creation of resources, and ownership over resources. It would not have been possible to develop these resources without the unique expertise gained through the lived experiences of this group, further demonstrating the crucial need to include lived experience voices in research and design (Dudgeon et al., 2018; McAlister et al., 2017).

The decision was made to focus on the co-creation of a Fact Sheet resource. This decision was made because (1) the Aboriginal community members identified this resource would be useful, and (2) of budget constraints, restricting our ability to deliver additional in-person media training across communities. Given the preliminary positive evidence of this training, future research should seek to build upon the programme developed/owned by M.K., in line with our recommendations. This training should be empowering, flexible and place-based, so that it can adapt to the needs and context of each local community.

An important future direction will be to co-design and run similar training across diverse communities. A limitation of the current study is that we were only able to run the training on Noongar Country, although our results establish the foundation for future training sessions with different community groups. Another limitation of our study was that the results were only initially analysed by one non-Indigenous researcher. However, several steps were taken to ensure the cultural validity of the results, including feedback loops with the Aboriginal media expert, senior researcher, and community members, as part of the APAR methodology.

The Australian National Partnership Agreement on Closing the Gap, commits to *‘listen to the voices and aspirations of Aboriginal and Torres Strait Islander people and change the way we work in response’*. (Clause 19: Commonwealth of Australia, 2020). To achieve this, Aboriginal and Torres Strait Islander voices (especially grassroot community members) must be given opportunities to engage in truth-telling. How the media reports, interprets and communicates about Aboriginal and Torres Strait Islander mental health challenges and suicide, can have further impacts on Aboriginal and Torres Strait Islander mental health and suicide. For example, media which perpetuates explicit and implicit racial biases against Aboriginal and Torres Strait Islander peoples, negatively impacts health and wellbeing (Dudgeon et al., 2021; Fogarty et al., 2018; Larson et al., 2007). The media also influences how the wider society view issues impacting Aboriginal and Torres Strait Islander peoples. In Australia, we have seen the power of the media in shaping stories and attitudes towards political issues impacting Aboriginal and Torres Strait Islander peoples, including the recent Voice to Parliament (Muller, 2023).

## Conclusion

For effective and accurate media reporting, Aboriginal and Torres Strait Islander voices must be included in public narratives about issues impacting Aboriginal and Torres Strait Islander peoples. The media has been responsible for perpetuating negative racial stereotypes, breaking cultural protocols, and reporting in biased ways on these issues. These actions have negative impacts on mental health and wellbeing. Breaking through these barriers requires a proactive consideration of how Aboriginal and Torres Strait Islander voices can be given more space in the media, including how to build the capability of Aboriginal and Torres Strait Islander peoples to share their truth in a way which is safe for them. Our set of recommendations and co-created resources will help facilitate Aboriginal and Torres Strait Islander truth-telling about mental health challenges and suicide to the media.

## Supplemental Material

sj-pdf-1-anp-10.1177_00048674251328542 – Supplemental material for Truth-telling about suicide: Empowering Aboriginal and Torres Strait Islander people to engage with the media Supplemental material, sj-pdf-1-anp-10.1177_00048674251328542 for Truth-telling about suicide: Empowering Aboriginal and Torres Strait Islander people to engage with the media by Pat Dudgeon, Jemma Collova, Gerry Georgatos, Ee Pin Chang, Elizabeth Paton, Mel Benson and Megan Krakouer in Australian & New Zealand Journal of Psychiatry

## Notes

1. *A First Nations guide for truth-telling about suicide (CBPATSISP & Everymind)*: https://cbpatsisp.com.au/wp-content/uploads/2024/04/QRC_Aboriginal-Torres-Strait-Islander_FINAL_APR_2024.pdf
