# Supplementary material for: Truth-telling about suicide: Empowering Aboriginal and Torres Strait Islander people to engage with the media
Source: Aust N Z J Psychiatry. 2025 Mar 27;59(5):448–56. doi: 10.1177/00048674251328542 (PMC12022366; doi:10.1177/00048674251328542)
Supplement: sj-pdf-1-anp-10.1177_00048674251328542 – Supplemental material for Truth-telling about suicide: Empowering Aboriginal and Torres Strait Islander people to engage with the media [file sj-pdf-1-anp-10.1177_00048674251328542.pdf]

Appendix 1. Survey (pre and post)

We want to understand how much experience you have had reporting on Aboriginal and Torres Strait Islander mental health or suicide to media. Please tick what applies below.

- ☐ I have spoken to media multiple times about mental health or suicide
- ☐ I have spoken to media once about mental health or suicide
- ☐ I have been approached by the media to speak, but chose not to speak to media
- ☐ I have no experience speaking to media about mental health or suicide

Please respond to the following questions on a scale of 1 (not at all) to 7 (very)

Do you feel you know how to engage with media to talk about mental health or suicide?

1 - 2 - 3 - 4 - 5 - 6 - 7

Not at all Somewhat Very

How likely would you be to engage with media to talk about mental health or suicide?

1 - 2 - 3 - 4 - 5 - 6 - 7

Not at all Somewhat Very

How confident do you / would you feel talking to media about mental health or suicide?

1 - 2 - 3 - 4 - 5 - 6 - 7

Not at all Somewhat Very

What would be your biggest worry about talking to media about mental health and suicide?

___

___

<u>Complete the following once you have finished the training:</u>

Do you feel you know how to engage with media to talk about mental health or suicide?

1 - 2 - 3 - 4 - 5 - 6 - 7

Not at all Somewhat Very

How likely would you be to engage with media to talk about mental health or suicide?

1 - 2 - 3 - 4 - 5 - 6 - 7

Not at all Somewhat Very

How confident do you / would you feel talking to media about mental health or suicide events/issues?

1 - 2 - 3 - 4 - 5 - 6 - 7

Not at all Somewhat Very

How useful did you find this training and why?

____

____

____

Did the facilitator (Megan) effectively assist in your learning and knowledge? What was good about them?

____

____

____

Appendix 2: Group Yarning – guide

Q 1. Did you feel the training was culturally appropriate? What made it so? (for example, was it safe for Aboriginal and Torres Strait Islander peoples, was the learning style appropriate, was the duration sufficient)?

Q 2. What was the most valuable thing you learnt or will take away from this training?

Q 3. Is there anything else you would like to learn, or that could have been improved in this training?

Q 4. How do you think your community will benefit from you engaging with media?

Q 5. Who else would benefit from this training?

Q 6. What do you want to (or not want to) see in media reports about mental health and suicide?

Q 7. How has this training helped you?

Q 8. What extra resources would you like to take away after this training?

Q 9. Did the media training enhance your knowledge, attitudes and perceptions of Aboriginal and Torres Strait Islander mental health and suicide? How?
